# To Investigate the Clinical Efficacy and Potential Mechanism of Tongxinluo Capsules in Preventing Coronary Restenosis Based on Meta-Analysis and Network Pharmacology Analysis

**DOI:** 10.1155/2023/7985459

**Published:** 2023-01-27

**Authors:** Wen-Xin Wei, Yi-Hao Jiang

**Affiliations:** ^1^Queen Mary College, Nanchang University, Nanchang, Jiangxi, China; ^2^School of Resources, Environment and Chemical Engineering, Nanchang University, Nanchang, Jiangxi, China

## Abstract

**Objective:**

The aim of this study is to evaluate the clinical efficacy of Tongxinluo capsules in preventing coronary restenosis based on meta-analysis and network pharmacology research methods and to preliminarily explore its intervention mechanism.

**Methods:**

First, through meta-analysis, we comprehensively searched databases such as CNKI, Wanfang, PubMed, the Cochrane Library, and Web of Science to find out the randomized controlled trials of Tongxinluo capsules in the treatment of coronary restenosis until February, 2022. According to the Cochrane Library, risk bias assessment tools were used to evaluate the included literature and Review Manager 5.2 software was used to conduct statistical analysis of the included studies. Then, based on network pharmacology, through TCMSP database and BATMAN-TCM database screening, the chemical components of Tongxinluo capsules and their related effects, symptom, and common targets were analyzed. String net was used to construct protein-protein interaction (PPI) network, and R3.6.1 software was used to carry out GO biological process enrichment analysis and KEGG signaling pathway enrichment analysis to clarify key pathways.

**Results:**

The meta-analysis finally included 10 RCTs with a total of 1318 subjects. Meta-analysis results showed that Tongxinluo capsules combined with conventional cardiovascular drugs could significantly improve the clinical efficacy of preventing in-stent restenosis and the clinical efficacy of preventing angina pectoris. There was no significant difference in the clinical efficacy of preventing myocardial infarction. Network pharmacology obtained a total of 101 chemical components and 149 targets through the online database. The results of network analysis showed that the targets were mainly involved in receptor ligand activity, carboxylic acid binding, steroid hormone receptor activity, and other related action pathways and were also involved in AGE-RAGE signaling pathway, cell senescence signaling pathway, and other related pathways.

**Conclusion:**

Tongxinluo capsules combined with conventional cardiovascular drugs can improve the clinical efficacy of preventing in-stent restenosis and angina pectoris and have a significant effect on reducing inflammatory factors. The comprehensive result of the effect is mainly through the participation of receptor ligand activity, carboxylic acid binding, steroid hormone receptor activity, and other ways to achieve the purpose of treating coronary restenosis.

## 1. Introduction

With the improvement of Chinese people's material living standard and the change of lifestyle, the morbidity and mortality of coronary heart disease are increasing year by year, and it has become a major health hazard disease. Cardiovascular and cerebrovascular diseases (CVD) have caused serious health problems in China. According to relevant statistics, about 45 percent of patients died of CVD in 2019. About 2.5 million of them had coronary heart disease (CHD) and about 200,000 died [1]. It is estimated that there will be 11.39 million cases of coronary heart disease in China in 2022. Coronary heart disease is more common in the elderly, and coronary stenting percutaneous coronary intervention (PCI) is the main mode for the treatment of CHD [2, 3], with the advantages of rapid recovery and small surgical trauma [4, 5]. But studies have pointed out the existence of risk for stent restenosis after surgery [6]. Based on this, in recent years, combining traditional Chinese and Western medicine treatment for prevention and control of PCI in postoperative in-stent restenosis (ISR), thereby improving the quality of survival, has become the new exploration point.

Tongxinluo capsule is based on traditional Chinese medicine (TCM) collaterals epidemiology and under the guidance of the theory of ZangXiang doctrine developed by compound Chinese medicine, by ginseng, leech, scorpion, Peony root, cicada slough, terrapin, centipede, sandalwood, incense, frankincense, sour jujube kernel, and borneol, are composed of ten aftertaste Chinese herbs, which have the effects of nourishing blood, invigorating qi, promoting blood circulation, removing blood stasis, clearing collaterals, and relieving pain. Ginseng has the effect of replenishing qi; leeches, red peony root, terrapin, and frankincense have the effect of activating blood circulation; scorpions, cicada slough, and centipedes have the function of channeling channels and collaterals, sandalwood and incense have the function of qi. Jujube seed has the effect of nourishing blood; borneol has both the function of activating blood and channeling channels. Clinically, Tongxinluo capsules combined with conventional cardiovascular drugs have been proved to be effective in preventing coronary restenosis after PCI. Unfortunately, the mechanism is not clear.

This study investigated the clinical efficacy of Tongxinluo capsules combined with conventional cardiovascular drugs in preventing coronary restenosis after PCI through meta-analysis and preliminarily analyzed its target and possible pathway of action through network pharmacology to explore their intervention mechanisms, which provided a scientific basis for Tongxinluo capsules in preventing coronary restenosis.

## 2. Materials and Methods

### 2.1. Meta-Analysis

#### 2.1.1. Literature Inclusion Criteria

The literature inclusion criteria were as follows: (1) Randomized controlled trials (RCTs) published in China and abroad; (2) the language is limited to Chinese and English; and (3) the subjects were patients after PCI, that is, patients with angina symptoms confirmed by coronary angiography (CAG) as single or multiple vessel lesions (target vessel stenosis *I* > 70%) with coronary heart disease or myocardial infarction.

#### 2.1.2. Literature Exclusion Criteria

The literature exclusion criteria were as follows: (1) Inclusion criteria were not met; (2) animal experiments; (3) literature review; (4) literature with poor design and incomplete data; (5) repeated publications; and (6) patients with other complications (such as acute myocardial infarction).

#### 2.1.3. Evaluation Indicators

In-stent restenosis (ISR), angina pectoris (AP), myocardial infarction (MI), IL-6, and total incidence of adverse cardiovascular events.

#### 2.1.4. Literature Retrieval Strategy Databases Retrieved Include

China National Knowledge Infrastructure (CNKI, https://www.cnki.net/), Wanfang Journal Database (Wanfang, https://www.wanfangdata.com.cn), PubMed (https://pubmed.ncbi.nlm.nih.gov), the Cochrane Library (https://www.cochranelibrary.com/), and Web of Science (https://www.webofscience.com/). The search period was from the establishment of the database to February, 2022. Chinese keywords mainly included “coronary artery,” “restenosis,” “clinical medicine,” and “Tongxinluo capsule.” English search words mainly include “Tongxinluo capsule,” and “in-stent restenosis.”

#### 2.1.5. Data Extraction and Quality Assessment

All literature were screened according to inclusion and exclusion criteria, and relevant baseline information including title, author, year, intervention measures, and observation indicators were summarized for those literature that met the criteria. The quality of the included literature was evaluated according to the cochrane risk bias assessment tool, including: (1) generation of random sequences; (2) allocation hidden; (3) blind subjects or researchers; (4) data integrity; (5) whether there is selective reporting of results; and (6) other biases. According to the literature content, three grades of “low risk,” “high risk,” and “unbelievable risk” were carried out for the literature.

#### 2.1.6. Data Statistics and Analysis Review

Manager 5.2 software was used for data analysis, and heterogeneity assessment was made for the included literature according to the *P* value and *I*^2^ index of heterogeneity test. If there was homogeneity among studies (i.e., *P* ≥ 0.1 and *I*^2^ ≤ 50%), a fixed-effect model was used for analysis; otherwise, random effect model was used. Odds ratio (OR) and 95% confidence intervals (CI) were selected to describe the results. The control group was divided into “conventional treatment” and “conventional treatment + one drug” for subgroup analysis according to different intervention measures.

### 2.2. Network Pharmacology Methods

#### 2.2.1. Chemical Constituents of Tongxinluo Capsules

Chemical constituents of Tongxinluo capsules were collected through Traditional Chinese Medicine Systems Pharmacology Database and Analysis Platform (TCMSP, https://tcmspw.com/tcmsp.php); the Bioinformatics Analysis Tool for Molecular mechanism of Traditional Chinese Medicine database (BATMAN-TCM, https://bionet.ncpsb.org.cn/batman-tcm/) was used to search the chemical constituents of Tongxinluo capsules. The TCMSP database is screened based on oral bioavailability (OB) ≥ 30% and drug-like (DL) ≥ 0.18 in the absorption, distribution, metabolism, and excretion (ADME) parameters [7]. The BATMAN-TCM database is screened by predicting candidate targets (including known targets) with a score of no less than 20 points for each component, *P* ≥ 0.5 as the standard [8]. Cytoscape V3.6.1 software was used to construct the drug component-target network.

#### 2.2.2. Construction of Target Database to Components and Target Database Related to the Coronary Artery Restenosis Target Database

Construction of target database corresponding to components and target database related to coronary artery restenosis target database TCMSP and BATMAN-TCM database were used to retrieve the targets of each chemical component in Tongxinluo capsules, and the chemical components with relevant information were screened. Targets for each chemical component are standardized using the Search Tool for the Retrieval of Interacting Genes/Proteins database (STRING, https://string-db.org/). The targets of chemical components in Tongxinluo capsule were searched and the chemical components with relevant information were screened. The search term “coronary restenosis” in Online Mendelian Inheritance in Man database (OMIM, https://www.omim.org/) and GeneCards database (https://www.genecards.org/) to find and coronary artery restenosis related targets. Through Venny2.1 database (https://bioinfogp.cnb.csic.es/tools/venny), screening of component targets, disease targets, and the intersection of targets as potential targets was performed for Tongxinluo capsule for the treatment of coronary artery restenosis.

#### 2.2.3. Construction and Analysis of the Protein Interaction Network

The intersection targets were imported into STRING database to obtain the protein interaction network and save it in TSV format. The Node1, Node2, and combined score information in the file are imported to Cytoscape V3.6.1 software, and the “Network Analyser” function in Cytoscape software is used for topology analysis of protein-protein interaction (PPI) Network, taking degree value as reference. We calculate the “connectivity,” “closeness centrality (CC),” and “betweenness centrality (BC)” of all nodes to determine the key targets in the network.

#### 2.2.4. GO Biological Analysis and KEGG Pathway Analysis

R3.6.1 software was used for enrichment analysis of GO biological process and KEGG signal pathway, and histogram, bubble diagram, and pathway diagram were drawn for visualization. GO bioanalysis was explained and annotated from three aspects: biological process, molecular function, and cellular location.

## 3. The Results

### 3.1. Meta-Analysis Results

#### 3.1.1. Literature Retrieval and Inclusion Results

**Figure 1:**
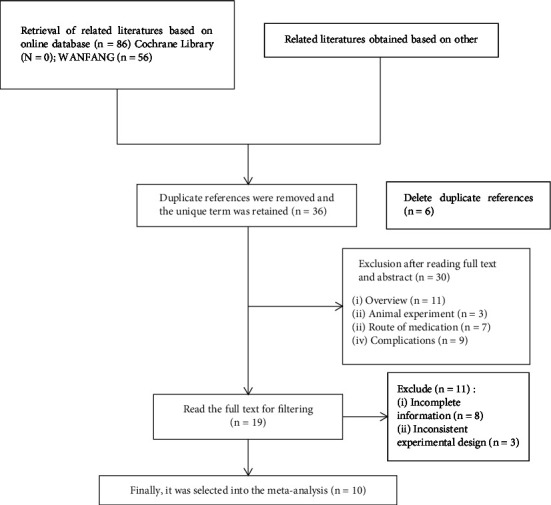
The specific process of literature screening.

Based on the above retrieval strategy, a total of 86 Chinese literature and 10 English literature were retrieved, and 36 literature were obtained after removing duplicates. After further screening, 10 Chinese literature were included, with a total of 1318 patients. The literature screening process and results are shown in Figure 1.

#### 3.1.2. Basic Features of the Included Literature

A total of 1318 patients were included in 10 studies, including 668 in the experimental group and 650 in the control group. The included basic characteristics are shown in Table 1.

#### 3.1.3. Risk Assessment of Bias in the Included Studies

**Figure 2:**
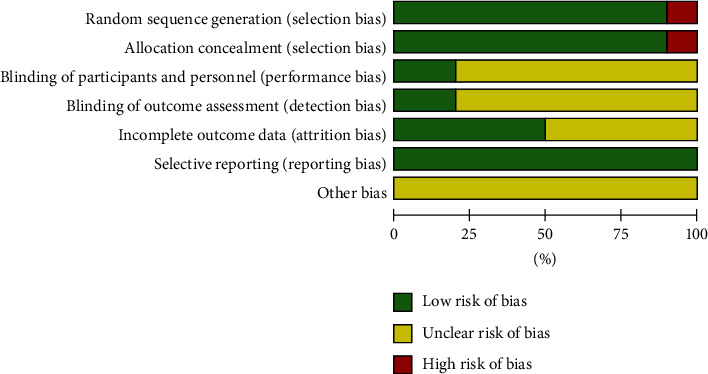
Risk of bias graph, review author's judgement about each risk of bias item presented as percentages across all included studies.

The Cochrane Risk Assessment Scale was used to assess the risk of bias and the quality of the 10 included articles was evaluated. In this study, 1 literature [7] was grouped according to the order of medical treatment and evaluated as “high risk,” while the other 9 literature mentioned random grouping and were all random number table and evaluated as “low risk.” Eight studies did not mention distribution hiding and blindness, which was evaluated as “unbelievable risk.” Two literature [10, 16] mentioned distribution hiding and blindness, which was evaluated as “low risk.” Five literature [7, 8, 10, 12, 13] did not clearly explain the data integrity and were evaluated as “unclear risk,” while the other five literature all had clear outcome indicators and were evaluated as “low risk.” No repeated publication or published bias was found in all studies, and the evaluation was “low risk.” Other bias is unknown and evaluated as “unclear risk.” The overall risk of bias for included studies is shown in Figure 2.

#### 3.1.4. Meta-Analysis Results

**Figure 3:**
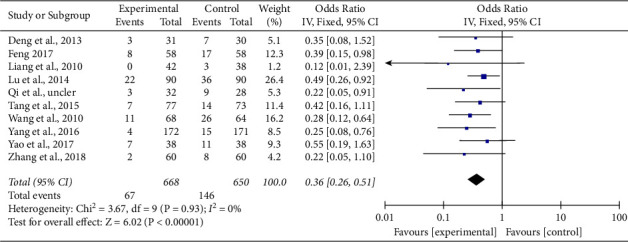
Forest plot of comparison of in-stent restenosis (ACS secondary preventive treatment + Tongxinluo capsules versus ACS secondary preventive treatment in ISR).

**Figure 4:**
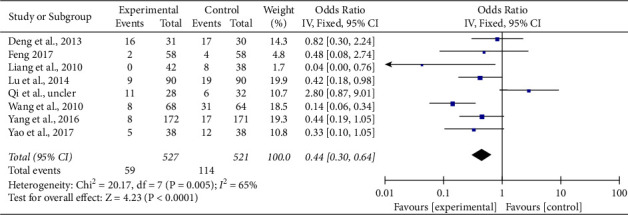
Forest plot of comparison of angina pectoris (ACS secondary preventive treatment + Tongxinluo capsules versus ACS secondary preventive treatment in AP).

*(1) Evaluation and Analysis of Prevention of ISR*. A total of 10 literature [7–16] reported the effective rate of clinical treatment for the prevention of in-stent restenosis, and the heterogeneity test result was (Chi^2^ = 3.50, DF = 9 (*P*=0.94), *I*^2^ = 0%). Due to high experimental homogeneity and zero experimental heterogeneity, the fixed-effect model was adopted. According to the results of meta-analysis (OR = 0.37, 95% CI (0.26, 0.51), *Z* = 5.97, *P* < 0.00001), the effective rate of the experimental group was significantly higher than that of the control group, and the frequency of ISR in the experimental group was significantly lower than that of the control group, with a statistical significance. Therefore, it can be considered that Tongxinluo capsules combined with conventional cardiovascular drugs can improve the clinical treatment efficiency of preventing ISR (See Figures 3 and 4).

*(2) Prevention of AP Analysis*. A total of 8 literature [7, 8, 10–14, 16] reported the clinical effectiveness of prevention of angina pectoris, and the heterogeneity test results were (Chi^2^ = 20.17, DF = 7 (*P*=0.005), *I*^2^ = 65%). Due to high heterogeneity, the random-effect model was adopted. According to the results of meta-analysis (OR = 0.44, 95% CI (0.30, 0.64), *Z* = 4.23, *P* < 0.0001), the effective rate of the experimental group was significantly higher than that of the control group, while the number of angina cases in the experimental group was significantly lower than that in the control group with a statistical difference. Therefore, it can be considered that Tongxinluo capsules combined with conventional cardiovascular drugs group can improve the clinical effective rate of prevention of angina pectoris (See Figure 4).

**Figure 5:**
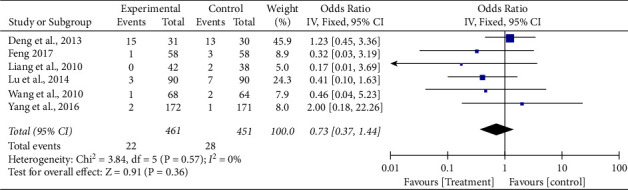
Forest plot of comparison of myocardial infarction (ACS secondary preventive treatment + Tongxinluo capsules versus ACS secondary preventive treatment in MI).

*(3) Analysis of Prevention of MI*. A total of 6 literature [8, 10–12, 14, 16] reported the clinical effectiveness of prevention of angina pectoris, and the heterogeneity test result was (Chi^2^ = 3.84, DF = 5 (*P*=0.57); *I*^2^ = 0%). Due to homogeneity, the fixed-effect model was adopted. According to the results of meta-analysis (OR = 0.73, 95 %CI (0.37, 1.44), *Z* = 0.91, *P* < 0.36), the results showed that the effective rate of the experimental group was not significantly higher than that of the control group, which was not statistically significant. Therefore, it is not considered that Tongxinluo capsules combined with conventional cardiovascular drugs can improve the clinical treatment efficiency of preventing myocardial infarction (See Figure 5).

**Figure 6:**
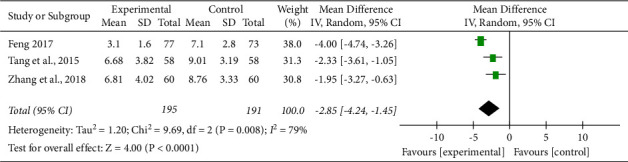
Funnel plot of comparison of IL-6 (ACS secondary preventive treatment + Tongxinluo capsules versus ACS secondary preventive treatment in IL-6).

*(4) IL-6 Level*. A total of 3 literature [9, 14, 15] reported the level of inflammatory factor IL-6, and the heterogeneity test result was ( Chi^2^ = 9.69, DF = 2 (*P*=0.008), *I*^2^ = 79%), indicating large heterogeneity, so the random effect model was adopted. According to the meta-analysis results (MD = −2.85, 95% CI (−4.24, −1.45), *Z* = 4.00, *P* < 0.001), there was a statistical difference between the treatment group and the control group. The level of IL-6 in the treatment group was significantly lower than that in the control group, which proved that Tongxinluo capsules had a good function of improving the IL-6 level (See Figure 6).

**Figure 7:**
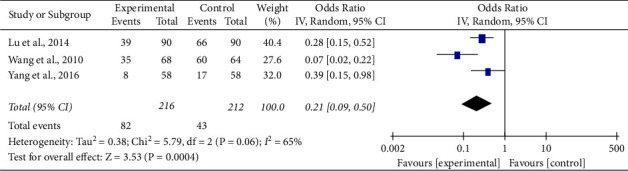
Funnel plot of comparison of TACE (ACS secondary preventive treatment + Tongxinluo capsules versus ACS secondary preventive treatment in TACE).

*(5) Incidence of Total Adverse Cardiovascular Events (TACE)*. A total of 3 literature [8, 10, 16] reported total adverse cardiovascular events, and the heterogeneity test result was (Chi^2^ = 5.86, *df* = 2 (*P*=0.05); *I*^2^ = 66%). Due to high heterogeneity, the random effect model was adopted. According to the results of meta-analysis (MD = 0.21, 95% CI (0.09, 0.50), *Z* = 3.50, *P*=0.0005), there was a statistical difference between the treatment group and the control group. The number of patients having adverse cardiovascular events in the treatment group was significantly lower than that in the control group, so Tongxinluo capsules can be considered to have a good therapeutic effect in adverse cardiovascular events (See Figure 7).

**Figure 8:**
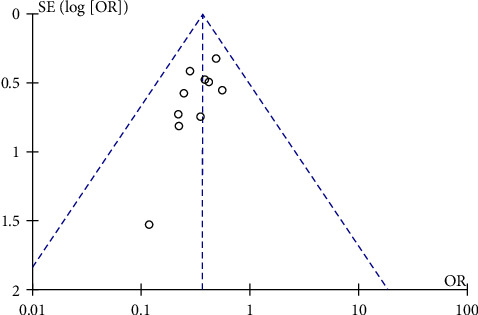
Funnel plot of comparison of the publication bias test.

*(6) Publication Bias Test Funnel Plots*. Publication bias test funnel plots were used to test a publication bias, an outcome measure of clinical efficacy in preventing ISR, showing asymmetrical distribution between studies, suggesting a high possibility of bias (Figure 8).

### 3.2. Pharmacological Results of the Network

#### 3.2.1. Chemical Constituents of Tongxinluo Capsules

Chemical constituents of Tongxinluo capsules were collected through TCMSP Database, the BATMAN-TCM database, and the chemical composition of database retrieval Tongxinluo capsules. The TCMSP database is screened based on oral bioavailability (OB) ≥ 30% and drug-like (DL) ≥ 0.18 in the ADME parameters [2]. The BATMAN-TCM database is screened by predicting candidate targets (including known targets) with a score of no less than 20 points for each component, with *P* ≥ 0.5 as the standard [3]. Cytoscape V3.6.1 software was used to construct drug component-target network.

#### 3.2.2. Construction of Target Database Corresponding to Components and Target Database

Construction of target database corresponding to components and target database related to coronary artery restenosis was performed. TCMSP database and BATMAN-TCM database were used to retrieve the targets of each chemical component in Tongxinluo capsules, and the chemical components with relevant information were screened. Targets for each chemical component are standardized using the STRING database. The targets of chemical components in Tongxinluo capsules were searched and the chemical components with relevant information were screened. The search term “coronary restenosis” in OMIM database (https://www.omim.org/) and GeneCards database (https://www.genecards.org/) to find coronary artery restenosis-related targets. Using Venny 2.1 database (https://bioinfogp.cnb.csic.es/tools/venny), we screened component targets and disease targets the intersection of targets, namely, for Tongxinluo capsules in the treatment of coronary artery restenosis potential targets.

#### 3.2.3. Construction and Analysis of the Protein Interaction Network

The intersection targets were imported into STRING database to obtain the protein interaction network and save it in TSV format. The Node1, Node2, and combined score information in the file are imported to Cytoscape V3.6.1 software, and the “Network Analyser” function in Cytoscape software is used for topology analysis of PPI Network, taking Degree value as reference. We calculate the “connectivity,” “closeness centrality (CC),” and “betweenness centrality (BC)” of all nodes to determine the key targets in the network.2.2.4 GO biological analysis, and KEGG Pathway Analysis R3.6.1 software was used for enrichment analysis of GO biological process and KEGG signal pathway, and histogram, bubble diagram, and pathway diagram were drawn for visualization. GO bioanalysis was explained and annotated from three aspects: biological process, molecular function, and cellular location.

### 3.3. Pharmacological Results of the Network

#### 3.3.1. Ingredient Screening of Tongxinluo Capsules

**Figure 9:**
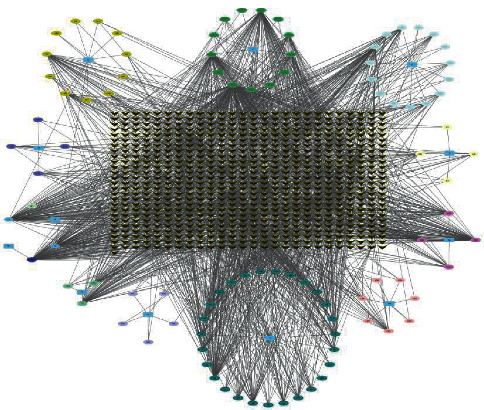
Drug-chemical-target network diagram of Tongxinluo capsules.

The ADME and prediction target component scores of Tongxinluo capsules were searched by TCMSP and BATMAN-TCM databases, and OB ≥ 30% and DL ≥ 0.18 were obtained. Score ≥ 20 and *P* ≥ 0.5 were the screening conditions. A total of 101 chemical components were retrieved after weight removal. See Table 2 for details. Drug component-target network diagram is shown in Figure 9.

### 3.4. Retrieval Results of Chemical Component Targets and Coronary Artery Restenosis Targets

The chemical targets of Tongxinluo capsules were obtained by searching TCMSP and BATMAN-TCM databases, and 678 targets were obtained after weight removal. A total of 763 disease targets were identified in GeneCards and OMIM databases using “coronary restenosis” as the key word. The drug action targets and disease targets were mapped and compared, and 149 intersection targets were obtained. The intersection results are shown in Figure 9.

### 3.5. Protein Interaction Network Construction and Analysis Results

**Figure 10:**
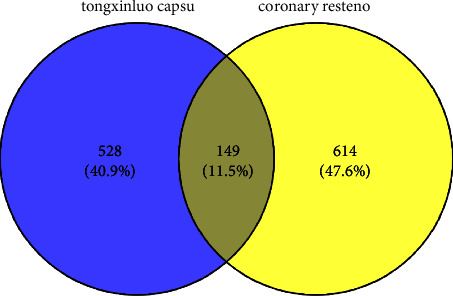
venn diagram of common target acquisition.

The common targets were input into STRING database, the species were defined as human, the protein interaction relationship was obtained, and the TSV format file was saved and imported into Cytoscape software for visualization. The results are shown in Figure 10, and the topology parameter analysis is shown in Table 3 (the first 30).

**Figure 11:**
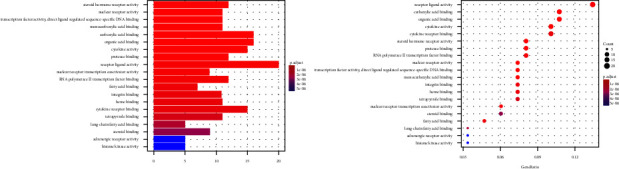
Molecular function analysis of common targets.

**Figure 12:**
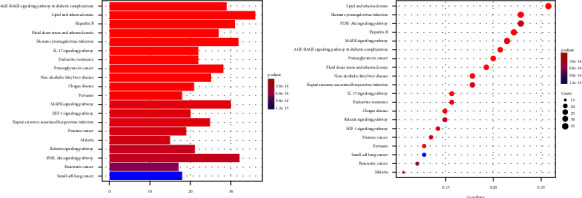
KEGG pathway analysis of common targets.

R3.6.1 software was used to conduct GO enrichment analysis on the intersection target of Tongxinluo capsules and coronary artery restenosis. “Receptor ligand activity,” “carboxylic acid binding,” “receptor ligand activity,” and 148 items including “steroid hormone receptor activity” (*P* < 0.05, Figure 11). KEGG pathway analysis found “Lipid and atherosclerosis,” “MAPK signaling pathway,” and “nonalcoholic fatty liver” disease and 180 related pathways. The result is shown in Figure 12.

## 4. Discussion

### 4.1. Significance of This Study

PCI is currently the main means for the treatment of acute coronary syndrome (ACS). Although PCI can effectively and quickly relieve the symptoms of patients, the incidence of postoperative coronary artery restenosis is still high, between 20% and 30%, accompanied by the occurrence of AP and MI, mentioned adverse cardiac and cerebrovascular events (MACCE), etc.

The Tongxinluo capsule is widely used in the treatment of cardiovascular diseases. Its main components are ginseng, borneol, leeches, centipede, turtle worm, peony root, etc., which have the effects of antiplatelet aggregation and antithrombosis and lowers blood lipids. Combination drug therapy can effectively reduce the incidence of restenosis after PCI.

Network pharmacology partial coronary restenosis ISR is a serious problem in patients undergoing PCI. In this study, the ADME and predictive target component scores of single drug in Tongxinluo capsules were searched through the advantages of network pharmacology, which is broad, multiaspect, and multitarget, to explore the possible mechanisms of active components, key targets, and potential pathways in the treatment process, so as to provide scientific basis for the treatment of coronary artery restenosis by Tongxinluo capsules.

### 4.2. Active Component Analysis

A total of 101 effective components related to Tongxinluo capsule in the treatment of coronary artery restenosis were identified. However, due to the complex composition of the Tongxinluo capsule, the core components in the treatment of coronary artery restenosis cannot be determined temporarily.

### 4.3. Mechanism Analysis

R3.6.1 software was used to conduct GO enrichment analysis on the intersection targets of Tongxinluo capsules and coronary artery restenosis. Through KEGG pathway analysis of common targets, it was found that the age-rage signaling pathway may indirectly improve diseases such as angiogenesis, inflammation, and atherosclerosis through a series of signal transduction by acting on AGEs molecules. It can promote the treatment of coronary artery restenosis with Tongxinluo capsules.

**Figure 13:**
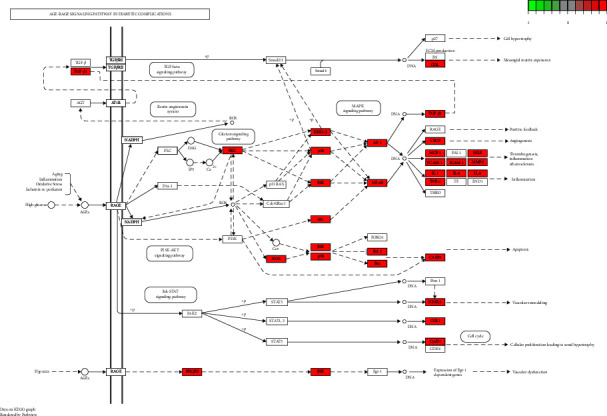
Network mechanism diagram of Tongxinluo capsule.

Among them, the age-rage signaling pathway has an obvious mechanism of action and ranks high in the signaling pathway. The Tongxinluo capsule may ameliorate coronary restenosis through the age-rage signaling pathway. The specific influence mechanism is as follows: aging, inflammation, oxidative stress, ischemia reperfusion, and other diseases can indirectly affect AGEs molecules, activate and promote RAGE genes, and indirectly affect PLC genes, thereby promoting DAG and IP3 molecules. DAG directly promotes PKC gene, while IP3 indirectly promotes calcium ion, and calcium directly promotes PKC gene. PKC gene indirectly acts on ERK1/2, P38, and JNK genes in three pathways, respectively. Erk1/2 and P38 indirectly act on AP-1, while P38 and JNK indirectly act on NF-*κ*B. It directly acts on VEGF, McP-1, TNF-*α*, IL-6, IL-1, and IL-8 genes, and ultimately indirectly improves angiogenesis, inflammation, and atherosclerosis (shown in Figure 13).

**Figure 14:**
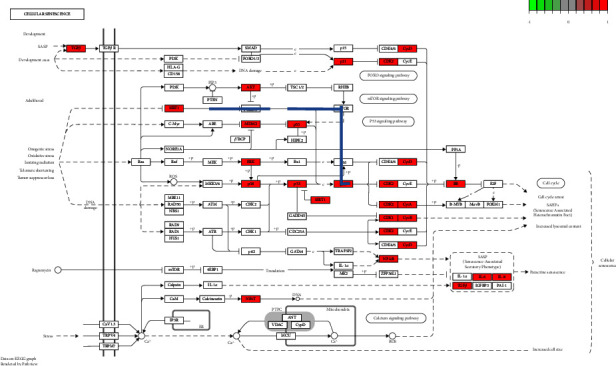
Network mechanism diagram of Tongxinluo capsules.

The improvement of coronary restenosis by Tongxinluo capsules may be related to the cellular senescence signaling pathway. When affected by external oxidative stress, ionizing radiation, telomere shortening, and loss of tumor inhibition, SIRT1 gene is indirectly inhibited, and FOXO3 gene is directly inhibited, p21 gene is directly promoted through ionization, CDK2 gene and CycE gene are directly inhibited, RB gene is directly inhibited by phosphorylation, and finally E2F gene is directly inhibited. Finally, cell cycle arrest, senescence related chromatin lesion, and lysosome content increase were indirectly affected, that is, cell senescence was ultimately affected (shown in Figure 14).

In addition, studies have found that cell senescence signals are also associated with coronary artery restenosis. When stimulated by external adverse factors, cell cycle arrest is indirectly affected, and senescence-related chromatin lesions and lysosome content are increased, that is, cell senescence is ultimately affected and coronary artery restenosis is promoted.

In summary, through this study, the main medicinal components of Tongxinluo capsules as well as its possible targets and related pathways were preliminarily clarified. The method is relatively complete and the screening results are supported by relevant literature. Subsequent cell or animal experiments on the predicted results are required, which provides ideas and directions for more subsequent studies.

### 4.4. Clinical Efficacy Analysis

The 10 studies included in this study analyzed the clinical efficacy of Tongxinluo capsules combined with conventional chemical therapy compared with conventional combined therapy in preventing the occurrence of coronary artery restenosis. Compared with conventional chemotherapy, Tongxinluo capsules combined with conventional chemotherapy has a better preventive effect and can effectively reduce the postoperative incidence of ISR, AP, IL-6, and total adverse cardiovascular events but has no significant effect on the postoperative prevention of MI.

### 4.5. Recommendations for Clinical Studies

This study has a certain reference value for the clinical treatment of coronary artery restenosis. The results of this study showed that Tongxinluo capsules combined with conventional chemical drugs had better effect than conventional combined drugs and no significant adverse reactions were observed. Therefore, in the future clinical treatment of coronary artery restenosis, Tongxinluo capsules can be used on the basis of conventional combined drugs according to patients' conditions to achieve a better therapeutic effect. In addition, it is suggested that researchers should clearly record the specific methods of blind uncovering and allocation of conceals in future clinical studies and carefully record various indicators and clinical trial reports to minimize the risk of deviation.

### 4.6. Deficiencies and Limitations of the Study

(1) Only two of the literature included in this study MACCE, so the sample size was insufficient to conduct effectiveness analysis; (2) the quality of the literature included in this study was uneven, only two literature clearly pointed out that the blind method was applied, so various biases might be generated; (3) a small number of research literature were included, resulting in a small sample size, which may produce bias; (4) the course of treatment and dose selected in the included literature are inconsistent, which may cause bias; (5) most of the included literature have few indicators and cannot be effectively analyzed; (6) network pharmacology is based on the results of network data and computational simulation and has not been verified by *in vitro* and *in vivo* experiments.

## 5. Conclusion

Based on the meta-analysis and network pharmacology research techniques, this study first evaluated the clinical efficacy of Tongxinluo capsules in treating ISR and analyzed its potential mechanism of action. The results showed that in 10 studies, the experimental group was significantly better than the control group in the treatment of ISR, AP, inflammatory cytokine IL-6, and the incidence of total adverse cardiovascular events. However, there was no significant difference in the treatment of MI between the control and treatment groups. A total of 101 effective components related to Tongxinluo capsules in the treatment of coronary artery restenosis were identified. There were 148 related pathways, mainly involving the age-rage signaling pathway and cell senescence signaling pathway, etc. Tongxinluo capsules may improve coronary artery restenosis through the age-rage signaling pathway and cell senescence signaling pathway.

## Figures and Tables

**Table 1:** Baseline characterization of included literature.

Included studies and year	Treatment	*Simple data*		Group method of data handling (GMDH)	*Age*		*Male*		*Intervening measure*		Blind method
		T	C		T	C	T	C	T	C	
Qi et al. 2012 [7]	6 M	32	28	Sequential order	Unclear	Unclear	Unclear	Unclear	①	②	Unclear
Lu et al. 2014 [8]	12 M	90	90	Random number table	60.2 ± 6.9	61.8 ± 7.2	53.30%	51.10%	①	②	Unclear
Tang et al. 2015 [9]	4 W	77	73	Random number table	53 ± 11	52 ± 10	59.70%	58.90%	①	②	Unclear
Wang et al. 2010 [10]	12 M	68	64	Random number table	65.2 ± 9.9	65.1 ± 10.4	63.20%	70.30%	①	②	Double blind
Liang et al. 2010 [11]	6 M	42	38	Random number table	Unclear	Unclear	Unclear	Unclear	①	②	Unclear
Deng et al. 2013 [12]	6 M	31	30	Random number table	59	59.5	67.70%	66.70%	①	②	Unclear
Yao et al. 2006 [13]	6 M	38	38	Random number table	56.6 ± 10.9	50.1 ± 12.4	Unclear	Unclear	①	②	Unclear
Feng et al. 2017 [14]	6 M	58	58	Random number table	61.02 ± 6.31	61.16 ± 6.34	67.2 0%	68.90%	①	②	Unclear
Zhang et al. 2018 [15]	6 M	60	60	Random number table	61.3 ± 5.5	62.4 ± 6.4	55.00%	Unclear	①	②	Unclear
Yang et al. 2016 [16]	6 M	172	171	Random number table	57.93 ± 9.77	58.63 ± 10.27	79.60%	78.30%	①	②	Double blind

T: experimental group, C: control group, ①: ACS secondary preventive treatment + Tongxinluo capsule, ②: ACS secondary preventive treatment.

**Table 2:** List of main chemical constituents of Tongxinluo capsules.

PubChem CID	Compounds	Molecular formula	Medicinal materials	Reference value
33934	Diop	C_24_H_38_O_4_	Ginseng	OB ≥ 30%, DL ≥ 0.18
5280794	Stigmasterol	C_29_H_48_O	Ginseng\radix paeoniae rubra	OB ≥ 30%, DL ≥ 0.18
222284	Beta-sitosterol	C_29_H_50_O	Ginseng\radix paeoniae rubra\rosewood heart wood	OB ≥ 30%, DL ≥ 0.18
91510	Inermin	C_16_H_12_O_5_	Ginseng	OB ≥ 30%, DL ≥ 0.18
5280863	Kaempferol	C_15_H_10_O_6_	Ginseng	OB ≥ 30%, DL ≥ 0.18
5319581	Scopolamine	C_16_H_12_NO_3_	Ginseng	OB ≥ 30%, DL ≥ 0.18
285342	Deoxyharringtonine	C_28_H_37_NO_8_	Ginseng	OB ≥ 30%, DL ≥ 0.18
441562	Dianthramine	C_14_H_11_NO_6_	Ginseng	OB ≥ 30%, DL ≥ 0.18
5312542	Arachidonate	C_20_H_32_O_2_	Ginseng	OB ≥ 30%, DL ≥ 0.18
441965	Frutinone A	C_16_H_8_O_4_	Ginseng	OB ≥ 30%, DL ≥ 0.18
119307	Ginsenoside rh2	C_36_H_62_O_8_	Ginseng	OB ≥ 30%, DL ≥ 0.18
—	Ginsenoside-Rh4_qt	C_36_H_60_O_8_	Ginseng	OB ≥ 30%, DL ≥ 0.18
96943	Girinimbine	C_18_H_17_N_O_	Ginseng	OB ≥ 30%, DL ≥ 0.18
73498	Panaxadiol	C_30_H_52_O_3_	Ginseng	OB ≥ 30%, DL ≥ 0.18
132350840	Suchilactone	C_21_H_20_O_6_	Ginseng	OB ≥ 30%, DL ≥ 0.18
—	Alexandrine_qt	C_35_H_60_O_6_	Ginseng	OB ≥ 30%, DL ≥ 0.18
4970	Fumarine	C_20_H_19_NO_5_	Ginseng	OB ≥ 30%, DL ≥ 0.18
5280445	Luteolin	C_15_H_10_O_6_	Sandalwood	OB ≥ 30%, DL ≥ 0.18
5281654	Isorhamnetin	C_16_H_12_O_7_	Sandalwood	OB ≥ 30%, DL ≥ 0.18
162350	Isovitexin	C_21_H_20_O_10_	Sandalwood	OB ≥ 30%, DL ≥ 0.18
72	Protocatechuic acid	C_7_H_6_O_4_	Cicada slough	OB ≥ 30%, DL ≥ 0.18
16072146	N-[(E)-2-[(2R,3S)-3-acetamido-2-(3,4-dihydroxyphenyl)-2,3-dihydro-1,4-benzodioxin-7-yl]vinyl]acetamide	C_20_H_20_N_2_O_6_	Cicada slough	OB ≥ 30%, DL ≥ 0.18
6437642	(3Z)-3-(2-amino-4,6-dioxo-5,8-dihydro-1H-pteridin-7-ylidene)-2-oxopropanoic acid	C_20_H_20_N_2_O_6_	Cicada slough	OB ≥ 30%, DL ≥ 0.18
8768	Protocatechualdehyde	C_7_H_6_O_3_	Cicada slough	OB ≥ 30%, DL ≥ 0.18
774	Histamine	C_5_H_9_N_3_	Centipede	Score ≥ 20, *P* ≥ 0.05
1153	Tyrosin	C_9_H_11_NO_3_	Centipede	Score ≥ 20, *P* ≥ 0.05
6274	L-Histidine	C_6_H_9_N_3_O_2_	Centipede	Score ≥ 20, *P* ≥ 0.05
5997	Cholesterol	C_27_H_46_O	Centipede/scorpio/woodlouse	Score ≥ 20, *P* ≥ 0.05
6106	Leucine	C_6_H_13_NO_2_	Centipede	Score ≥ 20, *P* ≥ 0.05
—	Stearin	C_21_H_42_O_4_	Scorpio	Score ≥ 20, *P* ≥ 0.05
5318035	20-Hexadecanoyl-Ingenol	—	Scorpio	Score ≥ 20, *P* ≥ 0.05
1123	Taurine	C_2_H_7_NO_3_S	Scorpio	Score ≥ 20, *P* ≥ 0.05
1146	Trimethylamine	C_3_H_9_N	Scorpio	Score ≥ 20, *P* ≥ 0.05
5281233	Crocin	C_44_H_64_O_24_	Leech	Score ≥ 20, *P* ≥ 0.05
24721095	Gardenoside	C_17_H_24_O_11_	Leech	Score ≥ 20, *P* ≥ 0.05
64945	Ursolic acid	C_30_H_48_O_3_	Leech	Score ≥ 20, *P* ≥ 0.05
107848	Geniposide	C_17_H_24_O_10_	Leech	Score ≥ 20, *P* ≥ 0.05
11850	Dulcitol	C_6_H_14_O_6_	Leech	Score ≥ 20, *P* ≥ 0.05
10508035	Gardnerilin A	C_35_H_66_O_8_	Leech	Score ≥ 20, *P* ≥ 0.05
6251	D-Mannitol	C_6_H_14_O_6_	Leech	Score ≥ 20, *P* ≥ 0.05
772	Enoxaparin	C_26_H_42_N_2_O_37_S_5_	Leech	Score ≥ 20, *P* ≥ 0.05
5281232	Crocetin	C_20_H_24_O_4_	Leech	Score ≥ 20, *P* ≥ 0.05
—	Heparin	C_26_H_42_N_2_O_37_S_5_	Leech	Score ≥ 20, *P* ≥ 0.05
772	Nadroparin	C_26_H_42_N_2_O_37_S_5_	Leech	Score ≥ 20, *P* ≥ 0.05
12600	D-Mannoheptulose	C_7_H_14_O_7_	Leech	Score ≥ 20, *P* ≥ 0.05
5459879	L-Galactoheptulose	C_7_H_14_O_7_	Leech	Score ≥ 20, *P* ≥ 0.05
3080632	Stigmast-7-en-3-ol	C_29_H_50_O	Radix paeoniae rubra	OB ≥ 30%, DL ≥ 0.18
—	(2R,3R)-4-methoxyl-distylin	C_16_H_17_O_7_	Radix paeoniae rubra	OB ≥ 30%, DL ≥ 0.18
173183	Campest-5-en-3beta-ol	C_28_H_48_O	Radix paeoniae rubra	OB ≥ 30%, DL ≥ 0.18
5281331	Spinasterol	C_29_H_48_O	Radix paeoniae rubra	OB ≥ 30%, DL ≥ 0.18
5363269	Ethyl oleate (NF)	C_20_H_38_O_2_	Radix paeoniae rubra	OB ≥ 30%, DL ≥ 0.18
64982	Baicalin	C_21_H_18_O_11_	Radix paeoniae rubra	OB ≥ 30%, DL ≥ 0.18
5281605	Baicalein	C_15_H_10_O_5_	Radix paeoniae rubra	OB ≥ 30%, DL ≥ 0.18
442534	Paeoniflorin	C_23_H_28_O_11_	Radix paeoniae rubra	OB ≥ 30%, DL ≥ 0.18
—	Paeoniflorgenone	C_18_H_22_O_6_	Radix paeoniae rubra	OB ≥ 30%, DL ≥ 0.18
5281855	Ellagic acid	C_14_H_6_O_8_	Radix paeoniae rubra	OB ≥ 30%, DL ≥ 0.18
9064	(+)-catechin	C_15_H_14_O_6_	Radix paeoniae rubra	OB ≥ 30%, DL ≥ 0.18
12303645	Sitosterol	C_29_H_50_O	Radix paeoniae rubra	OB ≥ 30%, DL ≥ 0.18
667495	(2R)-5,7-dihydroxy-2-(4-hydroxyphenyl)chroman-4-one	C_15_H_12_O_5_	Rosewood heart wood	OB ≥ 30%, DL ≥ 0.18
114829	DFV	C_15_H_12_O_4_	Rosewood heart wood	OB ≥ 30%, DL ≥ 0.18
821279	(2R)-7-hydroxy-5-methoxy-2-phenylchroman-4-one	C_16_H_14_O_4_	Rosewood heart wood	OB ≥ 30%, DL ≥ 0.18
336327	Medicarpin	C_16_H_14_O_4_	Rosewood heart wood	OB ≥ 30%, DL ≥ 0.18
373261	Eriodyctiol (flavanone)	C_15_H_12_O_6_	Rosewood heart wood	OB ≥ 30%, DL ≥ 0.18
5319494	(3R)-4′-Methoxy-2′,3,7-trihydroxyisoflavone	C_16_H_14_O_6_	Rosewood heart wood	OB ≥ 30%, DL ≥ 0.18
14353662	(3R)-3-(2,3-dihydroxy-4-methoxyphenyl)-7-hydroxychroman-4-one	C_16_H_14_O_6_	Rosewood heart wood	OB ≥ 30%, DL ≥ 0.18
14353660	(3R)-3-(2,3-dihydroxy-4-methoxyphenyl)chroman-7,8-diol	C_16_H_16_O_6_	Rosewood heart wood	OB ≥ 30%, DL ≥ 0.18
—	(3R)-7,2′,3′-trihydroxy-4-methoxyisoflavan	C_16_H_16_O_5_	Rosewood heart wood	OB ≥ 30%, DL ≥ 0.18
5319565	9-O-Methylcoumestrol	C_16_H_10_O_5_	Rosewood heart wood	OB ≥ 30%, DL ≥ 0.18
5319422	3′-Methoxydaidzein	C_16_H_12_O_5_	Rosewood heart wood	OB ≥ 30%, DL ≥ 0.18
182259	(−)-Vestitol	C_16_H_16_O_4_	Rosewood heart wood	OB ≥ 30%, DL ≥ 0.18
10019512	(3S)-7-hydroxy-3-(2,3,4-trimethoxyphenyl) chroman-4-one	C_18_H_18_O_6_	Rosewood heart wood	OB ≥ 30%, DL ≥ 0.18
—	4′,5',7-trimethyl-3-methoxyflavone	C_19_H_118_O_3_	Rosewood heart wood	OB ≥ 30%, DL ≥ 0.18
442768	Dalbergin	C_16_H_12_O_4_	Rosewood heart wood	OB ≥ 30%, DL ≥ 0.18
—	7-hydroxy-4′-methoxy-2′,5′-dioxo-4-[(3R)-2',7-dihydroxy-4′-methoxyisoflavan-5′-yl]isoflavone	C_32_H_127_O_9_	Rosewood heart wood	OB ≥ 30%, DL ≥ 0.18
28125525	Butin	C_15_H_12_O_5_	Rosewood heart wood	OB ≥ 30%, DL ≥ 0.18
127790	Duartin	C_18_H_20_O_6_	Rosewood heart wood	OB ≥ 30%, DL ≥ 0.18
10065329	Isoduartin	C_18_H_20_O_6_	Rosewood heart wood	OB ≥ 30%,
442792	4-Hydroxyhomopterocarpin	C_17_H_16_O_5_	Rosewood heart wood	OB ≥ 30%, DL ≥ 0.18
23259932	(6aR,11aR)-3,9,10-trimethoxy-6a,11a-dihydro-6H-benzofurano [3,2-c]chromen-4-ol	C_18_H_18_O_6_	Rosewood heart wood	OB ≥ 30%, DL ≥ 0.18
23259933	(6aR,11aR)-3,9-dimethoxy-6a,11a-dihydro-6H-benzofurano[3,2-c] chromene-4,10-diol	C_17_H_16_O_6_	Rosewood heart wood	OB ≥ 30%, DL ≥ 0.18
32990315	Odoricarpin	C_18_H_18_O_6_	Rosewood heart wood	OB ≥ 30%, DL ≥ 0.18
5321142	Sativanone	C_17_H_16_O_5_	Rosewood heart wood	OB ≥ 30%, DL ≥ 0.18
5321501	Stevein	C_16_H_12_O_5_	Rosewood heart wood	OB ≥ 30%, DL ≥ 0.18
44446897	Vestitone	C_16_H_14_O_5_	Rosewood heart wood	OB ≥ 30%, DL ≥ 0.18
44446884	Violanone	C_17_H_16_O_6_	Rosewood heart wood	OB ≥ 30%, DL ≥ 0.18
5280551	Xenognosin B	C_16_H_12_O_5_	Rosewood heart wood	OB ≥ 30%, DL ≥ 0.18
14077830	(6aR,11aR)-9,10-dimethoxy-6a,11a-dihydro-6H-benzofurano [3,2-c]chromen-3-ol	C_17_H_16_O_5_	Rosewood heart wood	OB ≥ 30%, DL ≥ 0.18
5280378	Formononetin	C_16_H_12_O_4_	Rosewood heart wood	OB ≥ 30%, DL ≥ 0.18
101257	Tirucallol	C_30_H_50_O	Frankincense	OB ≥ 30%, DL ≥ 0.18
—	O-acetyl-*α*-boswellic acid	C₃₀H₄₈O₃	Frankincense	OB ≥ 30%, DL ≥ 0.18
—	3alpha-Hydroxy-olean-12-en-24-oic-acid		Frankincense	OB ≥ 30%, DL ≥ 0.18
9847548	Boswellic acid	C_30_H_46_O_4_	Frankincense	OB ≥ 30%, DL ≥ 0.18
520687	Phyllocladene	C_20_H_32_	Frankincense	OB ≥ 30%, DL ≥ 0.18
160487	(S)-Coclaurine	C_17_H_19_NO_3_	Spina date seed	OB ≥ 30%, DL ≥ 0.18
5742590	Daucosterol	C_35_H_60_O_6_	Spina date seed	OB ≥ 30% DL ≥ 0.18,
222284	Phytosterol	C_29_H_50_O	Spina date seed	OB ≥ 30%, DL ≥ 0.18
14729078	Sanjoinenine	C_29_H_35_N_3_O_4_	Spina date seed	OB ≥ 30%, DL ≥ 0.18
124034	Swertisin	C_22_H_22_O_10_	Spina date seed	OB ≥ 30%, DL ≥ 0.18
102063083	Zizyphusine	C_20_H_24_NO_4_^+^	Spina date seed	OB ≥ 30%, DL ≥ 0.18
64971	Mairin	C_30_H_48_O_3_	Spina date seed	OB ≥ 30%, DL ≥ 0.18
119034	Asiatic acid	C_30_H_48_O_5_	Borneol	OB ≥ 30%, DL ≥ 0.18

**Table 3:** Topology parameter analysis of protein interaction (top 30).

Gene	Connectivity	Intermediate centrality	Closeness centrality	Topological parameter
TNF	44.81000	0.043298	0.740000	0.311181
IL-6	45.12121	0.040793	0.740000	0.311181
ALB	44.48485	0.078305	0.743719	0.304691
AKT1	46.58763	0.038277	0.736318	0.319093
INS	46.39130	0.047215	0.718447	0.317749
TP53	49.39080	0.026911	0.694836	0.342992
VEGFA	51.53012	0.012023	0.678899	0.360350
IL1B	50.09756	0.017848	0.678899	0.347900
PTGS2	50.69512	0.025018	0.678899	0.352049
JUN	51.80247	0.018249	0.675799	0.359739
EGFR	50.87342	0.015414	0.666667	0.355758
CASP3	53.14103	0.011855	0.663677	0.371616
MMP9	52.60256	0.012034	0.657778	0.373068
PPARG	50.66234	0.022659	0.663677	0.351822
FOS	52.02740	0.027691	0.654867	0.358810
CXCL8	53.74286	0.007951	0.637931	0.378471
ESR1	53.15942	0.011699	0.640693	0.369163
NOS3	49.56522	0.049894	0.637931	0.346610
CCL2	55.28788	0.005754	0.629787	0.386629
IGF1	55.68182	0.008738	0.629787	0.389383
CCND1	54.86154	0.007079	0.619247	0.391868
ICAM1	56.01563	0.004671	0.621849	0.394476
SIRT1	53.71429	0.012423	0.621849	0.375624
MAPK8	55.42857	0.005980	0.624473	0.384921
MMP2	57.70492	0.005082	0.609054	0.412178
CAT	53.2459	0.033342	0.616667	0.374856
ACE	49.7500	0.028737	0.616667	0.345486
MAPK1	51.67797	0.009489	0.611570	0.361384
